# Review state-of-the-art of output-based methodological approaches for substantiating freedom from infection

**DOI:** 10.3389/fvets.2024.1337661

**Published:** 2024-03-14

**Authors:** Eleftherios Meletis, Beate Conrady, Petter Hopp, Thibaut Lurier, Jenny Frössling, Thomas Rosendal, Céline Faverjon, Luís Pedro Carmo, Jaka Jakob Hodnik, László Ózsvári, Polychronis Kostoulas, Gerdien van Schaik, Arianna Comin, Mirjam Nielen, Tanja Knific, Jana Schulz, Sabina Šerić-Haračić, Christine Fourichon, Inge Santman-Berends, Aurélien Madouasse

**Affiliations:** ^1^Department of Public and One Health, School of Health Sciences, University of Thessaly, Karditsa, Greece; ^2^Department of Veterinary and Animal Sciences, Faculty of Health and Medical Sciences, University of Copenhagen, Copenhagen, Denmark; ^3^Complexity Science Hub Vienna, Vienna, Austria; ^4^Norwegian Veterinary Institute, Ås, Norway; ^5^Université de Lyon, INRAE, VetAgro Sup, UMR EPIA, Marcy l’Etoile, France; ^6^Université Clermont Auvergne, INRAE, VetAgro Sup, UMR EPIA, Saint-Genès-Champanelle, France; ^7^Department of Disease Control and Epidemiology, National Veterinary Institute (SVA) Uppsala, Uppsala, Sweden; ^8^Department of Animal Environment and Health, Swedish University of Agricultural Sciences, Skara, Sweden; ^9^Ausvet Europe, Lyon, France; ^10^Veterinary Public Health Institute, Vetsuisse Faculty, University of Bern, Bern, Switzerland; ^11^Clinic for Reproduction and Large Animals-Section for Ruminants, Veterinary Faculty, University of Ljubljana, Ljubljana, Slovenia; ^12^Department of Veterinary Forensics and Economics, University of Veterinary Medicine Budapest, Budapest, Hungary; ^13^Royal GD, Deventer, Netherlands; ^14^Department of Population Health Sciences, Unit Farm Animal Health, Faculty of Veterinary Medicine, Utrecht University, Utrecht, Netherlands; ^15^Institute of Food Safety, Feed and Environment, Veterinary Faculty, University of Ljubljana, Ljubljana, Slovenia; ^16^Institute of Epidemiology, Friedrich-Loeffler-Institut, Federal Research Institute for Animal Health, Greifswald, Germany; ^17^Faculty of Veterinary Medicine, University of Sarajevo, Sarajevo, Bosnia and Herzegovina; ^18^Oniris, INRAE, BIOEPAR, Nantes, France

**Keywords:** freedom-from-infection, prevalence, output-based, surveillance, infectious-diseases

## Abstract

A wide variety of control and surveillance programmes that are designed and implemented based on country-specific conditions exists for infectious cattle diseases that are not regulated. This heterogeneity renders difficult the comparison of probabilities of freedom from infection estimated from collected surveillance data. The objectives of this review were to outline the methodological and epidemiological considerations for the estimation of probabilities of freedom from infection from surveillance information and review state-of-the-art methods estimating the probabilities of freedom from infection from heterogeneous surveillance data. Substantiating freedom from infection consists in quantifying the evidence of absence from the absence of evidence. The quantification usually consists in estimating the probability of observing no positive test result, in a given sample, assuming that the infection is present at a chosen (low) prevalence, called the design prevalence. The usual surveillance outputs are the sensitivity of surveillance and the probability of freedom from infection. A variety of factors influencing the choice of a method are presented; disease prevalence context, performance of the tests used, risk factors of infection, structure of the surveillance programme and frequency of testing. The existing methods for estimating the probability of freedom from infection are scenario trees, Bayesian belief networks, simulation methods, Bayesian prevalence estimation methods and the STOC free model. Scenario trees analysis is the current reference method for proving freedom from infection and is widely used in countries that claim freedom. Bayesian belief networks and simulation methods are considered extensions of scenario trees. They can be applied to more complex surveillance schemes and represent complex infection dynamics. Bayesian prevalence estimation methods and the STOC free model allow freedom from infection estimation at the herd-level from longitudinal surveillance data, considering risk factor information and the structure of the population. Comparison of surveillance outputs from heterogeneous surveillance programmes for estimating the probability of freedom from infection is a difficult task. This paper is a ‘guide towards substantiating freedom from infection’ that describes both all assumptions-limitations and available methods that can be applied in different settings.

## 1 Introduction

Many countries have regulations to control, eradicate, and sometimes prevent the introduction of infectious cattle diseases that are zoonotic or result in considerable economic losses, such as tuberculosis, bluetongue, foot and mouth disease, *Salmonella* Dublin (1–8). Furthermore, there are other cattle infections, such as bovine viral diarrhoea (BVD) or infectious bovine rhinotracheitis/infectious pustular vulvovaginitis (IBR/IPV), where there may be no regulatory requirement for eradication (9–12), that can cause substantial economic losses, due to suboptimal production and treatment costs, as well as decreased welfare in infected herds (13–19). Some countries claim to be free from BVD, whilst in others the infection is endemic (20, 21), hence the prevalence of major cattle diseases varies between countries.

In an effort to (i) limit the risk of introduction of infectious cattle diseases in infection-free areas and (ii) allow safe animal trading without restriction from countries that can prove freedom from infectious cattle diseases both World trade Organisation and EU have established rules based on the World Organisation for Animal Health guidelines (22) and regulations at EU level on the movements of live animals, respectively. EU regulations and/or legislations are often expressed as input-based standards. In detail, countries have to implement specific activities (i.e., inputs) such as surveillance strategies including pre-described design, sampling scheme, and type of tests to achieve an output, such as confidence in freedom from disease according to the regulation (23). For non-EU-regulated cattle diseases, member states and private organisations can implement control programmes based on country-specific conditions such as priority settings, availability of financial resources, epidemiological situations (e.g., prevalence of disease) and importance of export for the national economy. This results in a wide heterogeneity in the design and outcomes of control programmes. Consequently, prevalence levels or confidence in freedom from disease (when claimed) can be difficult to compare. This lack of comparability in the outcomes of heterogeneous control programmes can cause difficulties for intra-community trade, as livestock trade can lead to introduction of infectious agents into countries that are free from a specific disease (11).

To address this difficulty, it is possible to design surveillance programmes and analyse the surveillance data so that the outcomes are comparable, even though the surveillance modalities differ and are adapted to each context. This is referred as output-based surveillance. The basic principle of output-based standards is to define what has to be achieved in terms of confidence in freedom and not in terms of surveillance effort (24). To reach the same level of confidence in freedom from infection, it is not necessary to meet a detailed list of predefined requirements or to use the same methods for surveillance (23, 25).

When substantiating freedom from disease, the question is not to quantify the frequency of an event, but to estimate the likelihood of its absence, when there is no evidence of its presence. This is akin to problems addressed in other areas, such as demonstrating eradication of an invasive species (26–28) or confirming interruption in the transmission of human parasitic (29, 30) or infectious diseases (31, 32). In veterinary epidemiology, initial work on this question focused on determining an appropriate sample size to ensure some predefined level of confidence in disease freedom assuming a homogeneous population and a perfect biological test (33). Subsequent work improved on these initial assumptions by accounting for test imperfections (34), the clustering of animals within farms (35), and complex surveillance systems (36, 37). More recently, epidemiological models accounting for population structure, infection dynamics, and test imperfection have been developed (38–40). Four decades after the initial work on this question and given the ongoing interest in output-based surveillance for freedom from infection, an overview of output-based methodological approaches is still needed.

Thus, the objectives of this article are twofold. First to outline the epidemiological and methodological considerations when designing a surveillance programme in an output-based framework. The second objective is to provide an overview of the state-of-the-art methodological approaches for substantiating freedom from disease, that are basically the most common methods that are used or have the potential to be used for the evaluation of output-based surveillance for freedom from disease. Cattle infectious diseases are the focus, however examples from other species are mentioned. When the word disease is used in this article, as is often the case in the literature on the subject, it should be understood to mean infection by the agent causing the disease, whereas often surveillance programmes based on serological testings.

Section 2 summarises the epidemiological and methodological considerations for substantiating freedom from infection. Section 3 provides a review of existing output-based methodological approaches for substantiating freedom from infection, whilst sections 4—Discussion and 5—Conclusion, discuss the main finding of the review and provide study’s main conclusions, respectively. Supplementary material illustrates the differences in surveillance modalities with examples on BVD and *Mycobacterium avium subspecies paratuberculosis* (MAP).

## 2 Epidemiological and methodological considerations for substantiating of freedom from infection

### 2.1 Case definition

Main concern when dealing with an infectious disease is ensuring that animals do not harbour infectious agents that could be introduced and subsequently spread in a region/country. According to Christensen et al. (41) without a clear case definition, it is difficult to become free of any disease. A precise case definition, where the presence and detection of it leads to the loss of the free status, is needed. Nonetheless, a broader definition of a case such as that a case is an animal infected in the time interval for which freedom from infection is claimed, allows countries a more flexible designing of surveillance programmes. Another option is to include in the case definition, the population of interest which may be restricted to a production type in a farmed animal species, or refer to truly serological positive results or, at the other extreme, include all susceptible species and the environment. When implementing output-based surveillance, the objective is to assess the probability of freedom from infection of a country/region/herd, whereby surveillance results are from past activities and influence actions yet and in the future including choice of method for measurement of disease frequency (42). According to Vanderstichel et al. (43), the following four aspects have to be predefined in order to clearly the objectives of the surveillance activities: (i) the case definition (i.e., the criteria which qualify a positive case), (ii) the reference population, (iii) the period, and (iv) the design prevalence.

### 2.2 Substantiating freedom from infection as a statistical problem

Proving that a population is free from a given infection (at a given time) with absolute certainty requires that all the individuals from this population are tested with a perfect test (no false positive and no false negative test results). If all the individuals test negative, the population can be declared free from infection. However, neither a perfect diagnostic test exists nor testing the entire population at a given time is possible and thus an absolute proof of freedom is impossible to reach. Therefore, substantiating freedom from infection will be based on a sample from the population and for that reason there will always be some uncertainty, that must be quantified. For example, when determining presence or absence of disease the binomial distribution quantifies the probability of sampling any number of infected animals, assuming a certain disease prevalence in the population. The probability of having exactly k infected individuals in a particular sample, if π is the disease prevalence, n the number of individuals sampled and X the number of infected, is:


p(n,π)∼Binomial(n,π)

The question that the methods for substantiating freedom from disease address is the probability of obtaining a false negative surveillance outcome, i.e., P(X=0|n,π>0).

Therefore, estimating the level of confidence that a population is free from infection given a certain sampling scheme requires making the hypothesis that the true prevalence is greater than 0. This prevalence is chosen such that if the infection were present, the prevalence would be at least at this level. The chosen prevalence used as a reference is called the design prevalence, $\pi_{t}$. According to this assumption, it becomes possible to construct a test of hypothesis that can be statistically evaluated. The population is considered free if the probability of not detecting the infection, if it were present at the design prevalence level, is smaller than a chosen level of confidence. Following Heisey et al. (44) we define null and alternative hypotheses as follows:


$$H_{O}:\pi \geq \pi_{t}$$

$$H_{A}:\pi <\pi_{t}$$

Posing these hypotheses permits the precise definition of the two possible types of error associated with wrongly rejecting or accepting the null hypothesis. Table 1 summarises the possible outcomes of surveillance given whether the true disease prevalence is below or above the design prevalence and the associated type I (1-Sensitivity) and type II (1-Specificity) errors.

**Table 1:** Surveillance outcome as a function of disease status.

	True disease status	
	Disease is present	Disease is absent
Surveillance outcome	H_0_: π ≥ π_t_	H_A_: π < π_t_
Disease is present	Sensitivity of surveillance	Type II error
Disease is absent	Type I error	Specificity of surveillance

As explained by Heisey et al. (44) posing disease presence as the null hypothesis allows to base sample size calculations on a chosen Type I error probability which is equivalent to basing sample size calculations on the sensitivity of surveillance. By looking at this problem in terms of hypothesis testing and probability of error, it is possible to define standards for output-based surveillance to substantiate disease freedom.

### 2.3 Output-based surveillance standards

In an output-based framework, the amount of uncertainty regarding the true situation should be comparable across programmes. Cameron (45) described three generations of output-based standards: surveillance sensitivity/specificity, probability of freedom from infection and expected cost of error.

#### 2.3.1 Surveillance sensitivity and specificity

Surveillance sensitivity (SSe) represents the probability of detecting the infection given that it is truly present: SSe = P(O^+^ | D^+^). O^+^ and O^−^ are the surveillance outcome (respectively disease detected and not detected) and D^+^ and D^−^ the true disease situation (respectively disease is present and absent). An increase in the sensitivity of surveillance is associated with a decrease in the probability of falsely concluding that the infection is absent when it is in fact present, i.e., false negative results.

Surveillance specificity (SSp) is the probability of not detecting the disease given that it is truly absent: SSp = P(O^−^ | D^−^). SSp is most often assumed to be perfect (i.e., equal to 1) when substantiating freedom from infection. The reason is that when an infection is absent or rare, any positive test result will be investigated (diagnostic follow-up) until either proven to be a false positive or confirmed positive. The assumption of perfect specificity might not be reasonable if the disease is endemic or if no confirmatory tests are done.

#### 2.3.2 Probability of freedom from infection

Probability of freedom from infection (PFI) is the probability that an animal that comes from a herd or a country that claims to be free from infection is indeed free from infection, given the surveillance programme in place. It can also be formulated as what is the probability of freedom from infection given that the output of the surveillance programme is negative (i.e., the infection was not detected). This measure is basically the Negative Predictive value of the surveillance programme (SNPV): SNPV = P(D^−^ | O^−^). Table 2 explains how these measures relate to the outcome of the surveillance system and the assumption of disease presence or absence.

**Table 2:** Joint probabilities of disease status and surveillance outcomes.

	True disease status	
	Disease is present	Disease is absent
Surveillance outcome	P(D^+^) = π_t_	P(D^−^) = 1 − π_t_
Disease is present	SSe * π_t_	(1 − SSp) * (1 − π_t_)
Disease is absent	(1 − Sse) * π_t_	SSp * (1 − π_t_)

#### 2.3.3 Expected cost of error

The notion of expected cost of error could be a tool to economically justify the level of performance desired for a surveillance program. The expected cost of error weighs the probabilities of false positive and false negative results by the costs of their expected consequences. In a surveillance programme in which the probability of a false positive is null (specificity is equal to 1), the expected cost of error corresponds to the consequences of maintaining an undiagnosed infection in a herd/region/country or introducing an infected animal in an infection-free population. In this case, the expected cost of error can be expressed as the probability of a false negative output multiplied by its economic consequences (Cost_error_). The total cost of the surveillance programme (TSC) is equal to the sum of the surveillance costs (Cost_surv_) and the expected cost of error (ECE). The economically optimal surveillance programme corresponds to a trade-off between a surveillance programme with a higher or lower SSe. Indeed, a higher SSe implies generally a higher Cost_surv_ but a higher SNPV corresponding to a lower ECE. Conversely, a lower SSe implies generally a lower Cost_surv_ but a higher ECE. This approach was used to compare two different pork meat inspection components (visual only and traditional) for the surveillance of bovine tuberculosis in pork in Denmark (46). Further, Rout et al. (28) suggested not only to consider in the declaration of a successful eradication, the associated probability that eradication has been successful but also the cost of the programme itself, shown in management of foxes.

### 2.4 Assessing infection-free status: utilising surveillance data

Estimating a probability of freedom from infection in a country that is actually free from infection has to be based on simulations from hypotheses. That is to say that the method used for the estimation must (i) represent the disease as being present at the design prevalence, with a prevalence that can be higher in some strata of the population and lower in others and (ii) include different sampling intensities in different surveillance components as well as tests with different sensitivities. But, because the surveillance data are expected to contain only true negatives or false positives, no epidemiological knowledge can be gained from these data, except perhaps on the specificity of the different tests. On the other hand, when the disease is present in parts of the population and the objective is to identify units that are free, the methods used can estimate quantities that are relevant for the identification of infected units such as strength of association with risk factors and test characteristics. Heisey et al. (44) and Madouasse et al. (47) describe Bayesian models that estimate the strengths of association between risk factors and probabilities of infection from historical data. These estimates are then incorporated in the prediction of freedom from infection.

### 2.5 Factors determining the performance of surveillance systems for substantiating freedom from infection

In a surveillance system for substantiating freedom from infection it is assumed that: (i) the diagnostic method used perfectly differentiates infected from healthy individuals, (ii) all individuals in the population have the same probability of being infected, and (iii) all individuals in the population had the same probability of being tested. However, these are three assumptions that are usually not valid. Departure from these assumptions can result in difficulties when analysing the results of surveillance but can also be seen as an opportunity to improve the performance of surveillance, for example by preferentially testing the individuals with a higher probability of being diseased, known as risk-based sampling.

#### 2.5.1 Performance of the tests used for surveillance

Tests to detect a disease can sometimes be positive in uninfected individuals (false positives) or be negative in infected individuals (false negatives). The performance of a given test for the detection of a given infection is measured by its Sensitivity (*Se*) and Specificity (*Sp*). *Se* (i.e., probability of a positive test result given that the infection is present) and *Sp* (i.e., probability of a negative test outcome given that the infection is absent) are conditional probabilities and quantify the probability of a test outcome given an infection status. The main challenge is that, although individual animals are tested, what is of interest is the probability that the herd or the entire territory is free from infection. For example, when the disease has never been detected in a herd, an antibody positive test is indicative of a more or less recent presence of the infection. But in herds where recent measures have been taken to remove infected animals, the detection of antibody-positive individuals can reflect immunity to the pathogen without any infectious individual being present. In this case, test *Sp* for detecting infection at the herd level may be low. Low *Sp* at the herd level can lead to unnecessary and potentially costly measures, such as culling of animals, quarantine measures, or additional testing.

#### 2.5.2 Design of the surveillance system: surveillance components

An important feature of livestock farming is the structure of the population into farms that are themselves structured into sub-groups (e.g., barns, age-groups). When the infection is present in the region/country, the probability of infection is usually different between farms as well as between sub-groups within farms. On the other hand, when the region/country is infection free, there is a certain probability of introducing the infection which might differ between farms and/or between sub-groups within farms. When designing a risk-based surveillance system, maximising the sensitivity of detection, it is important to concentrate surveillance efforts on sub-groups where there is a higher probability of detecting the infection if it is present.

#### 2.5.3 Design of the surveillance system: frequency of testing

The frequency at which testing is performed and probability of freedom evaluated can have an impact on the performance of the surveillance system. The lower the frequency, the longer between infection introduction and its detection. The frequency of testing also has an impact on the frequency with which the probability of disease freedom can be evaluated. Most of the models allow the accumulated information to be incorporated into this evaluation (47–49).

Regardless of the method used, infection freedom calculations usually require a considerable amount of data such as on test results, animal population, associated industry, risk factors for the introduction of infection, biosecurity measures, and existing surveillance programmes.

## 3 Existing methods with potential for output-based surveillance

This section provides a review of state-of-the-art methods that are used or have the potential to be used for the evaluation of output-based surveillance for freedom from infection.

### 3.1 Scenario tree modelling

Scenario tree analysis, described by Martin et al. (37) is the current reference quantitative method to estimate the probability of freedom from disease/infection in complex surveillance systems. A surveillance system consists of surveillance component(s) represented as a separate branch of the scenario tree. Scenario trees are represented by different type of nodes: (i) Category nodes represent factors dividing the surveillance system population into subsets with different probabilities of being infected. (ii) An infection node represents the infection status; the associated branch probabilities are derived from design prevalence. (iii) Detection nodes represent the detection of infection and are associated with test characteristics.

The two main outputs from the scenario tree model are (i) an estimate of the probability of detecting a positive unit (animal or herd) if the infection is present in the population above the design prevalence, the so-called surveillance sensitivity and (ii) the probability that the population is free from infection. Furthermore, accounting for the probability of introduction of infection, it is possible to estimate probability of freedom from infection in the next time steps. The main assumptions critical for the method’s applicability are (i) all final results from the surveillance system have to be consistent with country or zone freedom from infection and (ii) the overall specificity of the surveillance system is 100%. A final result is considered the test result after completion of any diagnostic follow-up, since usually any positive test result will be further investigated until either proven to be a false positive or confirmed positive. In the latter case, confirmed positive outcome, the claim of freedom from infection is violated. This whole process of repeated testing (sequential testing) reduces the probability of a false-positive output in the surveillance system, resulting in a surveillance specificity equal to 1.

The overall surveillance sensitivity is an aggregation of the component surveillance sensitivities. The surveillance component sensitivity (*SCSe*) is given by the following formula:


$$SCSe=1-{\left(1-p^{*}*Se^{unit}\right)}^{n}$$

This equation answers to ‘what is the probability of the surveillance system detecting at least one case at the design prevalence’. The term $p^{*}*Se^{unit}$ is the probability that a single unit passing through the surveillance system, where infection is present at the design prevalence (p*) gets detected. The second output of the scenario tree is the probability of freedom from infection, that is basically the negative predictive value of the surveillance component (SPNV), SNPV = P(D^−^ | O^−^). Scenario trees split the population into homogenous subpopulations (sub-groups) that have the same probability of being infected (same risk of infection), thus allowing incorporation of risk information.

The outputs of the scenario tree method in an ongoing surveillance system can be updated at the end of a time period, based on Bayes’ theorem, allowing use of historical surveillance data and incorporation of infection introduction in the population over time [see (36, 50)]. Since the method was established in 2007, there have been published studies from all continents and terrestrial and aquatic animals, production animals and wildlife (51–53). In cooperation with FAO, a guideline for using the method has been developed (54).

### 3.2 Bayesian belief networks

Whilst scenario trees have been established as the reference method to substantiate freedom from infection, when it comes to complex surveillance schemes (e.g., multiple surveillance components) they become difficult to implement. Thus, Hood et al. (55) proposed an alternative method to calculate (i) the probability of freedom of infection and (ii) the overall surveillance sensitivity where the scenario tree is represented as a Bayesian Belief Network (BBN). A BBN is a probabilistic graphical model (directed acyclic graph), where the nodes in the graph represent random variables and the edges/arcs that connect the nodes represent the relationships between the random variables. Basically, BBNs provide a simple way of applying Bayes’ theorem to complex problems, defining the joint probability distribution for a set of variables. The structure of a surveillance programme can be represented by a BBN (simple scenario trees can be easily ‘translated’ and represented in a BBN). Hood et al. (55) ‘converted’ and represented the scenario tree described by Martin et al. (36) as a BBN. The unit’s surveillance sensitivity simply is the probability of the network to acquire a positive result. BBNs allow to update the beliefs and tune the variables (e.g., switch to targeted sampling scheme). The component surveillance sensitivity is estimated using the ensemble of posterior probabilities derived from all processed units. BBNs are not widely known and have not been broadly implemented to substantiate freedom from infection, because they require a specific software that is not considered user-friendly. The STOC free model, that is described below (Section 3.5.), can be seen as a Bayesian network that represents the time dependence between longitudinal herd-level observations, but not the within-herd group structure.

### 3.3 Simulation models

Simulation models represent the presence or spread of diseases and their detection with a great flexibility in terms of the range and complexity of assumptions that can be included (1, 2). As scenario trees, these models rely on stochastic simulations (i.e., model is run for a certain number of iterations) to evaluate ‘as if’ scenarios. They can generate the same outputs as scenario tree models such as statistical (posterior) distributions for sensitivity of surveillance and probability of disease freedom, as well as additional ones such as the cost of testing. Consequently, simulation models can be seen as extensions of the scenario tree methodology that allows a more complex representation of surveillance programmes. Such a simulation model was developed by Meyer et al. (38) to evaluate different testing strategies for demonstrating freedom from infection by MAP in the Republic of Ireland. Rosendal et al. (40) also used a simulation model to evaluate surveillance strategies for monitoring the state of disease freedom of Sweden with respect to infections by MAP. Simulation models allow the comparison of different surveillance strategies under different scenarios of disease presence or disease emergence and spread.

### 3.4 Bayesian prevalence estimation methods accounting for population structure

Bayesian models have been described for quantifying the probabilities of pest-eradication in feral-pigs from Santa-Cruz island, California (27), fox eradication, as an invasive predator, on Phillip Island in Australia (28). Modelling the diversity of surveillance programmes in an output-based framework may result in overparameterization if the structure of the population is not taken into account. Cattle populations can be considered structured in the following four basic levels: country/region/herd/animal. Thus, the joint probability distribution can be modelled to reflect the structure dependence. For instance, Heisey et al. (44) developed a Bayesian approach, adjusting for available covariate information, using surveillance data to substantiate freedom from infection on chronic wasting disease in deer. Generally, approaches for prevalence estimation and substantiation of freedom from infection at the country level, assuming presence or absence of perfect test(s), involve a multiple-stage cluster-sampling (35). In this setting a random number of k herds are selected and then a random sample of n animals from each herd are tested with one or more diagnostic methods. Bayesian methods for prevalence estimation and designation of a country’s infectious status, assuming imperfect *Se* and *Sp* of the applied test(s) have been described soon after 1998 (56–59). The developed methodologies account for the full structure of the population and allow estimation of the probability of freedom from infection at each level and test characteristics. The individual infectious status is modelled as latent (i.e., unknown but through probabilistic estimates inference can be made for it) (60). That type of model can be adapted to both disease presence and absence contexts because information on the true infectious status for every individual in every level is made available. However, it is up to decision making, how many positive animals declare an infected herd or how many positive herds declare a region not free. Usually, the metric that is used is the probability that the infection does not exceed a pre-specified critical level of 5% (58). Bayesian hierarchical modelling, adjusting for the population structure, allows implementation of complicated multi-level probability specifications (58).

### 3.5 STOC free model

The aim of the STOC free model (surveillance tool for outcome-based comparison of freedom from infection) is to predict herd-level probabilities of (freedom from) infection from longitudinal surveillance data (47, 48). It is a Bayesian Hidden Markov Model (HMM) in which true herd-level statuses regarding disease are modelled as latent binary variables with monthly dynamics. The latent statuses are inferred/predicted from successions of test results based on hypotheses regarding herd level test characteristics and infection dynamics. Risk factors of new infection can be included in the model when available. Estimation is performed in a Bayesian framework that allows the available knowledge of test characteristics, disease dynamics as well associations between risk factors and probability of new infection to be incorporated into the model as prior distributions. The prediction of the statuses of all herds in the data is performed for the last month of test results. Data available before this month are used as historical data for parameter estimation. The model is available as an R package that can be installed from GitHub.^1^ The main model outputs are posterior distributions for the probability of being diseased in each herd on the last month of surveillance. The model is suited to contexts where the infection is present, where all the participating herds are tested on a regular basis and when the objective is to identify infected from uninfected herds in the surveillance programme. It allows the inclusion of risk factors of new infections into the predictions. This model was used to estimate the probabilities of infection in herds considered as free from infection in four European countries (49).

One difficulty with the STOC free model is the need to obtain prior distributions for herd level test characteristics, i.e., probabilities of getting positive/negative test results given the true herd level status regarding infection. The methodological difficulties are not only restricted to the STOC free model.

## 4 Discussion

The most salient feature of estimating a probability of freedom from infection from surveillance data is that it generally involves quantifying the evidence of absence from the absence of evidence. In practical terms, this means that, in most cases, the infection that is sought to be proven absent has not been detected. The methodological difficulty is that the infection could be present but not detected for reasons related to imperfect test *Se* or sampling strategies.

Selecting a method for quantifying probability of freedom from infection relies on the assumed level of prevalence of infection. When infection is absent from an area, the objective is to prove that it has not been introduced to secure trade with partners outside the area. The level of interest is in this case the whole area. The scenario tree methodology and more recent simulation methods are well suited to this context (36, 37, 40). When the infection is still endemic, as in the initial phase of an eradication programme, the objective is to identify herds that are free from the infection within the programme to secure trade within and outside the area. In this case, historical data from the surveillance programme can be used to make inference and enhance the identification of infected herds. The STOC free model was designed to operate in this context (47).

A historical perspective on the methods developed for quantifying the probability of freedom from infection is of interest to understand the state-of-the-art. Early work focused on determining a sample size to prove that the infection prevalence was not greater than a chosen design prevalence with a certain level of confidence. This was initially done assuming homogeneous populations, in which all the animals had a similar probability of being infected, and a single perfect test was performed for detection of infection. These assumptions were later relaxed by considering the imperfect *Se* and *Sp* of the tests and the fact that animals are usually clustered within farms (57, 61). A later refinement was the inclusion of differences in the probability of infection between different animals or herds allowing for the estimation of probabilities of freedom from infection from surveillance systems that relied on risk-based sampling (54). Risk-based sampling permits to increase the effectiveness of surveillance by focusing surveillance efforts on areas where the infection is more likely to be found. The scenario tree method was designed to estimate a probability of freedom from infection and surveillance sensitivity from complex surveillance data with differences in the probability of infection in different components of the surveillance system (risk-based sampling) as well as imperfect sensitivity of the testing procedure (imperfect test sensitivity, sampling, hierarchical structure of the data with animals nested within farms). This, together with the fact that simulations could be run on spreadsheets led to this method being widely used for substantiating freedom from infection (62). More recent simulation models use the same principles as the scenario tree methodology.

The impact of imperfect *Sp* is not highlighted, because any positive test result will be investigated (diagnostic follow-up) until either proven to be a false positive or confirmed positive. In this sense, the proportion of false positives is reduced to 0, yielding a perfect Sp.

When the infection is absent from the area under investigation, it must be (re-) introduced to be present. Typically, an emergence should be a rare event with people making efforts to prevent the introduction of the infection through the routes of emergence perceived as important. Incorporating a probability of introduction into a model of infection freedom is therefore a difficult task. It is almost impossible to estimate a probability of introduction from data, as there are no or not enough similar cases of disease introduction in similar contexts. In this case, probabilities can be conceived as beliefs, which may have a rational basis, about the probability of introduction. The first view represents a frequentist perspective on probabilities and the second a Bayesian perspective. This is important to reflect upon when designing models and communicating their outcome. More generally, when the infection is absent, the whole estimation process relies on simulating its presence or its introduction under different scenarios, and out of these, count the proportion of times this infection would be detected by the surveillance system. Therefore, all the methods considered can be seen as conceptualising the problem of substantiating freedom from infection as a Bayesian problem in which what is evaluated is the probability of the hypotheses, notably in the form of a design prevalence, given the surveillance data collected. This explains why in some studies, the probability of the infection being present before collecting the data is referred to as the prior and the estimated probability of infection freedom as the posterior (36).

When the infection is still endemic, data from infected herds can be used to estimate strengths of association between risk factors and the probability of infection, thereby improving the detection of infection in herds not yet detected. This is the approach followed in the Bayesian models proposed by Heisey et al. (44) and Madouasse et al. (55). By exploiting the correlation in longitudinal test results, the model by Madouasse et al. (55) also estimates the herd-level *Se* and *Sp* of the tests used in the surveillance programme as well as the monthly probabilities of getting and eliminating the infection. By making inference from surveillance data, these models are less reliant on hypotheses whose validity can be hard to assess and provide predictions that are adapted to the context in which surveillance is performed. Such models can also produce knowledge that is transferable to other surveillance systems. However, when the infection is absent or rare, there is no added value to these Bayesian inference and prediction models since in those cases, they will perform simulations from the prior distributions used as input.

The definition of what is an infected herd can also be difficult to formulate. Returning to the example of infection by BVD, there are different types of infected animals that do not pose the same epidemiological risk and that do not react in the same way to the different tests. Persistently infected (PI) animals are the main source of infection for other animals. They shed massive amounts of virus but do not produce antibodies. Transiently infected animals shed the virus for a few weeks and produce antibodies against the virus for many years. Unborn PI foetuses are epidemiologically important, but it is not currently possible to detect infection before birth. The definition of what makes an infected herd in a surveillance programme could include or exclude either of the latter two categories. Translating *Se* or *Sp* at the animal level into their equivalent at the herd level can therefore be a real challenge. The issue can be difficult to perceive when using latent class models. With these models, the latent class, that should correspond to the definition of what the epidemiologists mean by infected herds, will depend on the prior distributions used for the *Se* and the *Sp* of the different tests used. As these *Se*s and *Sp*s can be unknown and modelled using best guesses, the model may work with a latent class that is different from the intended definition of an infected herd. The same problem also exists with all simulation-based models that use sensitivities elicited from experts. However, it is impossible to detect because, in the worst case, the infection will emerge once and be missed which can always be considered compatible with a surveillance sensitivity that is lower than one. The definition of sensitivities and specificities at the herd level is therefore a research gap that needs to be filled. A modelling framework needs to be developed to estimate these parameters from animal data and the required data need to be identified.

The objective of any method that quantifies a probability of freedom from infection from surveillance data is to assist stakeholders in making decisions about which countries or herds to trade with. The output of such methods should therefore be understandable and usable by stakeholders. Following Cameron (45), the most reported outputs are the sensitivity of surveillance as well as the probability of freedom from infection, which can be easily obtained using the scenario tree method. The STOC free model returns a posterior distribution for the probability of infection which can be translated into a distribution for the probability of freedom from infection. However, translating statistical distributions into information that is usable by decision makers can be challenging. Further work on communicating the output of quantitative models to decision makers should address this question. In addition to the specific output from methods for assessment of disease freedom, there is also a need to put this result into context and to make the information that will support decision more complete by adding qualitative aspects. Guidelines for incorporating necessary information about surveillance attributes have been proposed, e.g., by RISKSUR (63) and AHSURED (64).

Outputs related to the failure costs of surveillance, especially the cost of declaring an infected country as free from infection, have been proposed. Although the cost of different surveillance programme designs can be estimated (38), the cost of the consequences of an undetected emerging infectious disease are harder to predict. A first step in this direction consists in estimating the time to detection and the size of the outbreak at detection using models incorporating population dynamics and disease spread as proposed by Rosendal et al. (40).

A final concern is the fact that when using any method, an assumption is that the available data accurately reflect what they are supposed to measure. This assumes an infrastructure that collects these data in a reliable way and that the modellers are aware of all the limitations of the data they have, and the outcomes of the model can relatively easily be interpreted to the ‘users’ (e.g., farm managers, traders, veterinary officials).

Most of the concepts and methods reviewed could be applied to other animal and plant species. Although a few studies in fields not related to cattle farming were included in this work [e.g., (27, 50)], most of the concepts and methods reviewed here could be applied to other animal and plant species to quantify probabilities of absence of specific diseases in these species or even absence of these species. There are many scientific publications on the problem of evaluating the probability of absence of a species after an eradication programme. Sometimes, these species are problematic because they were introduced in a new environment in which they caused significant damage (27, 28). At other times, the focus is on estimating the probability that a human disease has been eradicated following a vaccination campaign. Although the problems may be different, the focus of these studies is on quantifying the evidence of absence from the absence of evidence and could therefore provide interesting ideas for such problems in cattle, and more broadly in livestock.

## 5 Conclusion

This review provides an overview of the epidemiological and methodological considerations for substantiating freedom from infection and existing output-based methodological approaches. It is evident that the process of substantiating freedom from infection based on surveillance data is an intricate task complicated by the potential failings of diagnostic tests and sampling methodologies.

Furthermore, this review aims to support researchers providing descriptions of the available output-based models and the described context in which the methods are most appropriate. Methods should be tailored in each case, for example in regions free of infection and regions where disease is still endemic. For areas claiming infection-free status, is needed to facilitate secure trading relationships, and scenario tree and simulation methods are commended for their efficacy in these circumstances. In contrast, for regions where infection is known to be present, particularly in the initial phases of an eradication effort, the focus shifts to identifying uninfected herds to safeguard trade and health both within the area and externally. The STOC free model, and generally Bayesian prevalence estimation methods, are highlighted for their effectiveness in this precise context.

Output-based methodological approaches for substantiating freedom from infection are signalling a significant step forward in managing infectious diseases, with implications for public and one health, economics, and the global community. The alliance of epidemiological considerations and methodological approaches forms the strategy towards control and eventually eradication of infectious diseases.

## Author contributions

EM: Conceptualization, Investigation, Writing – original draft, Writing – review & editing, Methodology. BC: Writing – review & editing, Writing – original draft. PH: Writing – review & editing. TL: Writing – review & editing. JF: Writing – review & editing. TR: Writing – review & editing. CFa: Writing – review & editing. LC: Writing – review & editing. JH: Writing – review & editing. LÓ: Writing – review & editing. PK: Writing – review & editing, Conceptualization. GS: Writing – review & editing. AC: Writing – review & editing. MN: Writing – review & editing. TK: Writing – review & editing. JS: Writing – review & editing. SŠ-H: Writing – review & editing. CFo: Writing – review & editing. IS-B: Writing – review & editing. AM: Conceptualization, Writing – original draft, Writing – review & editing.

## Footnotes

^1^ https://github.com/AurMad/STOCfree
