# Supplementary material for: Review state-of-the-art of output-based methodological approaches for substantiating freedom from infection
Source: Front Vet Sci. 2024 Mar 14;11:1337661. doi: 10.3389/fvets.2024.1337661 (PMC10977073; doi:10.3389/fvets.2024.1337661)
Supplement: Supplementary file 1 [file Data_Sheet_1.pdf]

Impact of the diversity of surveillance programmes on methods for output-based surveillance

For the same disease, there can be an important diversity in the design of surveillance programmes. This diversity can reflect vastly different epidemiological situations or can be the result of different choices in similar contexts. Even within a country, surveillance modalities can evolve over time to reflect changes in surveillance objectives because of the evolution in the epidemiological situation. These differences in surveillance modalities imply that either the methods used to quantify the probability of disease freedom can accommodate the resulting diversity in input or that different methods producing comparable outputs are used in different contexts. This is illustrated with examples on bovine viral diarrhea (BVD) and *Mycobacterium avium ssp. paratuberculosis* (MAP).

Several countries have eradicated BVD. The process of achieving national freedom from infection status can be divided into two parts: a pre-eradication phase, the objective of which is to identify infected herds and restrict the movement of their animals to stop the spread of infection, while at the same time implementing eradication measures in these herds to eliminate infection. When the infection prevalence is low enough the post-eradication phase can begin during which surveillance is conducted to prove freedom from infection to trading partners as well as to detect the infection early in case of re-emergence. For the eradication of BVD, countries have used different methods for identifying infected herds: testing all the animals (Presi et al., 2011), spot testing of calves (ear tags or blood sampling), testing the milk of primiparous cows or bulk tank milk testing to detect antibodies or the virus. Eradication is based on identifying and culling of persistently infected (PI) animals. Herds suspected of being infected are then retested and must perform regular surveillance and apply biosecurity measures to maintain their herd free status. Some countries additionally allow vaccination to

prevent reinfection of free herds. However, this limits the use of serological methods for BVD antibody detection (Houe et al., 2006).

Scandinavian countries started their BVD eradication programmes in the 90s (Houe et al., 2006). However, these countries had very different prevalence levels when starting their programmes. While Sweden and Denmark had a relatively high herd prevalence of BVD (40-50% of dairy herds), Norway and Finland started with prevalence levels of 7.1% and 1%, respectively (Houe et al., 2006). Since then, all these countries have achieved their goal of country level BVD eradication (Hodnik et al., 2021). Therefore, their current objective is to prove freedom from infection. All the programmes use different surveillance modalities for dairy and non-dairy herds. In dairy herds, bulk tank milk samples are tested, which is not possible in beef herds (Houe et al., 2006). The frequency of sample collection differs. For example, in Denmark every herd is sampled four times a year (Foddai et al., 2016) while in Norway only 12.5% of the herds are sampled on a yearly basis (Norström et al., 2014). Non-dairy herds are sampled at slaughter in abattoirs. In the case of positive results, the samples are retested and/or herd level investigations are conducted, either by spot testing young stock or a representative number of cattle in a herd (the number is dependent on herd size) (Houe et al., 2006). The seronegative or weakly positive animals are tested for BVD (Norström et al., 2014). Virus positive animals, if any, are culled and the herd is subjected to movement restriction until the last PI is removed. Monitoring of the farm status is conducted by spot testing of young stock or testing the milk of primiparous cows until the level of antibodies in adult animals drops (Houe et al., 2006). Most Scandinavian countries use the SVANOVIR™ BVD-Ab ELISA while Denmark uses a blocking ELISA which is less sensitive and specific for detecting antibodies in bulk tank milk (Foddai et al., 2014).

Paratuberculosis (PTBC) is a chronic contagious disease caused by *Mycobacterium avium subsp. paratuberculosis* (MAP). PTBC is an OIE-listed disease (OIE, 2021) and in a recent review of PTBC control programs in 48 countries, 73% of the studied countries recognized PTBC as a notifiable disease in dairy cattle and 46% of the reviewed countries had an established control program (Whittington et al., 2019). The infection is extremely hard to eradicate, notably because of the long delay between infection and clinical signs and the low sensitivity of the available tests. Therefore, in most countries in which the infection is endemic, the objective is to identify infected herds to control the infection in these herds. In this context, identifying herds that are free from infection is also important to protect them from introducing the infection as well as identifying them as safe to trade with (Hidano et al., 2016).

Most commonly, MAP control programs are based on a test-and-cull scheme, combined with breaking the disease transmission routes within and between herds (Garcia and Shalloo, 2015; Whittington et al., 2019). However, assessing the effectiveness of a PTBC control program, baseline data on the true prevalence of MAP infection is needed (Adaska and Anderson, 2003). Various methods are available for the detection of MAP infection, although no reference standard test with high sensitivity and specificity exists (Nielsen and Toft, 2009; Garcia and Shalloo, 2015). The gross and histopathological examination of cattle could provide a definitive diagnosis of PTBC but even this method is not perfect and may require a high number of samples to rule out infection (Whittington et al., 2019). Nevertheless, milk antibody ELISA tests, which are based on samples obtained during official milk recording, can be performed to assess MAP status (Meyer et al., 2018; Ózsvári et al., 2020), that is why ELISA testing is an integral part of many international PTBC testing and control programs (Kennedy et al., 2016).

In the different studies, herd-level and within herd animal-level true prevalence estimates vary widely, although, the direct comparison of the different MAP prevalence studies is difficult due to the differences in the applied diagnostic tests, testing strategies, and sampling design (Garcia and Shalloo, 2015). The Bayesian approach is a widely accepted method for assessing PTBC prevalence, and it enables a direct probabilistic interpretation of the estimates, moreover, range-respecting interval estimates can be obtained (i.e., prevalence estimates are within the range of 0 to 1) (Messam et al., 2008; Garcia and Shalloo, 2015). A Bayesian two-stage hierarchical model, which was constructed similarly to (Branscum et al., 2004), was used to estimate both herd- and animal-level PTBC prevalence in more recent studies (McAloon et al., 2016; Ózsvári et al., 2020).

In general, MAP infection is present in a relatively large proportion of the dairy herds, although, within certain herds, a relatively low proportion of the animals is infected (Muskens et al., 2000; Pozzato et al., 2011; Verdugo et al., 2015). According to the estimates provided by Nielsen and Toft, (2009), the herd-level true prevalence exceeds 50% and the within-herd animal-level true prevalence is higher than 3-5% in many countries. For instance, in Hungary, within the infected farms, the median animal-level true prevalence was 4.4% in primiparous and 10.3% in multiparous cows, respectively (Ózsvári et al., 2020). Herd-level true prevalence was estimated at 23 to 34% in Ireland (McAloon et al., 2016), around 70% in Italy (Pozzato et al., 2011), 75 to 92% in Denmark (Verdugo et al., 2015), and 89% in Hungary (Ózsvári et al., 2020). The herd-level true prevalence of PTBC varied from 2.8 to 91.1% in different studies, using a wide range of methods in various countries (Garcia and Shalloo, 2015). Within herd animal-level true prevalence estimates generally range from 2.7 to 15% ((Adaska and Anderson, 2003; Pozzato et al., 2011; Garcia and Shalloo, 2015; Verdugo et al., 2015; McAloon et al., 2016, Ózsvári et al., 2020). The Bayesian two-stage hierarchical model allows for the estimation of the probability of within-herd prevalence exceeding a pre-specified level

in the infected herds. This provides important insights for the design and implementation of a future control program that will be based on the discrimination between high, medium, and low risk herds.

Therefore, from a methodological point of view, the motivation for quantifying the probability of freedom from infection should evolve over time. In the eradication phase, it is important to identify infected from non-infected herds to limit movement from infected herds. When eradication has been achieved, proof that the whole territory is free from infection needs to be given to external trading partners. Given the context, the methods used should be able to incorporate surveillance data that differ in the level at which testing is performed (animal, batch, herd), the frequency of testing, the sensitivity and specificity of the tests used; accounting for the fact that this heterogeneity often lies within individual surveillance programmes.

References

Adaska, J.M., Anderson, R.J., 2003. Seroprevalence of Johne's-disease infection in dairy cattle in California, USA. Prev. Vet. Med. 60, 255–261. doi:10.1016/S0167-5877(03)00120-X

Branscum, A.J., I.A. Gardner, and W.O. Johnson. 2004. Bayesian modeling of animal-and herd-level prevalences. Prev. Vet. Med. 66(1-4):101-112. doi:10.1016/j.prevetmed.2004.09.009

Foddai, A., C. Enøe, K. Krogh, A. Stockmarr, and T. Halasa. 2014. Stochastic simulation modeling to determine time to detect Bovine Viral Diarrhea antibodies in bulk tank

milk. Prev. Vet. Med. 117:149–159. doi:10.1016/j.prevetmed.2014.07.007.

Foddai, A., A. Stockmarr, and A. Boklund. 2016. Evaluation of temporal surveillance system sensitivity and freedom from bovine viral diarrhea in Danish dairy herds using scenario tree modelling. BMC Vet. Res. 12:118. doi:10.1186/s12917-016-0744-2.

Garcia, A.B., and L. Shalloo. 2015. Invited review: The economic impact and control of paratuberculosis in cattle. J. Dairy Sci. 98:5019–5039. doi:10.3168/jds.2014-9241.

Hidano, A., T.E. Carpenter, M.A. Stevenson, and M.C. Gates. 2016. Evaluating the efficacy of regionalisation in limiting high-risk livestock trade movements. Prev. Vet. Med. 133:31–41. doi:10.1016/j.prevetmed.2016.09.015.

Hodnik, J.J., Ž. Acinger-Rogić, M. Alishani, T. Autio, A. Balseiro, J. Berezowski, L.P. Carmo, I. Chaligiannis, B. Conrady, L. Costa, I. Cvetkovikj, I. Davidov, M. Dispas, I. Djadjovski, E.L. Duarte, C. Faverjon, C. Fourichon, J. Frössling, A. Gerilovych, J. Gethmann, J. Gomes, D. Graham, M. Guelbenzu, G.J. Gunn, M.K. Henry, P. Hopp, H. Houe, E. Irimia, J. Ježek, R.A. Juste, E. Kalaitzakis, J. Kaler, S. Kaplan, P. Kostoulas, K. Kovalenko, N. Kneževič, T. Knific, X. Koleci, A. Madouasse, A. Malakauskas, R. Mandelik, E. Meletis, M. Mincu, K. Mõtus, V. Muñoz-Gómez, M. Niculae, J. Nikitović, M. Ocepek, M. Tangen-Opsal, L. Ózsvári, D. Papadopoulos, T. Papadopoulos, S. Pelkonen, M.P. Polak, N. Pozzato, E. Rapaliuté, S. Ribbens, J. Niza-Ribeiro, F.-F. Roch, L. Rosenbaum Nielsen, J.L. Saez, S.S. Nielsen, G. van Schaik, E. Schwan, B. Sekovska, J. Starič, S. Strain, P. Šatran, S. Šerić-Haračić, L.-M. Tamminen, H.-H. Thulke, I. Toplak, E. Tuunainen, S. Verner, Š. Vilček, R. Yildiz, and I.M.G.A. Santman-Berends. 2021. Overview of Cattle Diseases Listed Under Category C, D or E in the Animal Health Law for Which Control Programmes Are in

Place Within Europe. Front. Vet. Sci. 8:849. doi:10.3389/fvets.2021.688078.

Houe, H., A. Lindberg, and V. Moennig. 2006. Test Strategies in Bovine Viral Diarrhea Virus Control and Eradication Campaigns in Europe. J. Vet. Diagn. Invest. 18:427–436. doi:10.1177/104063870601800501.

Kennedy, A.E., N. Byrne, A.B. Garcia, J. O'Mahony, and R.G. Sayers. 2016. Analysis of Johne's disease ELISA status and associated performance parameters in Irish dairy cows. BMC Vet. Res. 12:43. doi:10.1186/s12917-016-0667-y.

McAloon, C.G., M.L. Doherty, P. Whyte, L. O'Grady, S.J. More, L.L.McV. Messam, M. Good, P. Mullowney, S. Strain, and M.J. Green. 2016. Bayesian estimation of prevalence of paratuberculosis in dairy herds enrolled in a voluntary Johne's Disease Control Programme in Ireland. Prev. Vet. Med. 128:95–100. doi:10.1016/j.prevetmed.2016.04.014.

Messam, L.L.M., A.J. Branscum, M.T. Collins, and I.A. Gardner. 2008. Frequentist and Bayesian approaches to prevalence estimation using examples from Johne's disease. Anim. Health Res. Rev. 9:1–23. doi:10.1017/S1466252307001314.

Meyer, A., K. Bond, S. Van Winden, M. Green, and J. Guitian. 2018. A probabilistic approach to the interpretation of milk antibody results for diagnosis of Johne's disease in dairy cattle. Prev. Vet. Med. 150:30–37. doi:10.1016/j.prevetmed.2017.11.016.

Nielsen, S.S., and N. Toft. 2009. A review of prevalences of paratuberculosis in farmed animals in Europe. Prev. Vet. Med. 88:1–14. doi:10.1016/j.prevetmed.2008.07.003.

Norström, M., M.E. Jonsson, J. Åkerstedt, A.C. Whist, A.B. Kristoffersen, S. Sviland, P. Hopp, and H. Wahlström. 2014. Estimation of the probability of freedom from Bovine

virus diarrhoea virus in Norway using scenario tree modelling. Prev. Vet. Med. 116:37–46. doi:10.1016/j.prevetmed.2014.06.012.

OIE. 2021. Paratuberculosis. Accessed October 25, 2021. https://www.oie.int/en/disease/paratuberculosis/.

Ózsvári, L., Zs. Lang, A. Monostori, P. Kostoulas, and I. Fodor. 2020. Bayesian estimation of the true prevalence of paratuberculosis in Hungarian dairy cattle herds. Prev. Vet. Med. 183:105124. doi:10.1016/j.prevetmed.2020.105124.

Pozzato, N., K. Capello, A. Comin, N. Toft, S.S. Nielsen, G. Vicenzoni, and N. Arrigoni. 2011. Prevalence of paratuberculosis infection in dairy cattle in Northern Italy. Prev. Vet. Med. 102:83–86. doi:10.1016/j.prevetmed.2011.07.001.

Presi, P., R. Struchen, T. Knight-Jones, S. Scholl, and D. Heim. 2011. Bovine viral diarrhea (BVD) eradication in Switzerland—Experiences of the first two years. Prev. Vet. Med. 99:112–121. doi:10.1016/j.prevetmed.2011.01.012.

Muskens, J., H.W. Barkema, E. Russchen, K. van Maanen, Y.H. Schukken, and D. Bakker. 2000. Prevalence and regional distribution of paratuberculosis in dairy herds in the Netherlands. Vet. Microbiol. 77:253–261. doi:10.1016/S0378-1135(00)00310-2.

Verdugo, C., N. Toft, and S.S. Nielsen. 2015. Within- and between-herd prevalence variation of Mycobacterium avium subsp. paratuberculosis infection among control programme herds in Denmark (2011–2013). Prev. Vet. Med. 121:282–287. doi:10.1016/j.prevetmed.2015.07.012.

Whittington, R., K. Donat, M.F. Weber, D. Kelton, S.S. Nielsen, S. Eisenberg, N. Arrigoni, R. Juste, J.L. Sáez, N. Dhand, A. Santi, A. Michel, H. Barkema, P. Kralik, P. Kostoulas, L. Citer, F. Griffin, R. Barwell, M.A.S. Moreira, I. Slana, H. Koehler, S.V. Singh, H.S. Yoo, G. Chávez-Gris, A. Goodridge, M. Ocepek, J. Garrido, K. Stevenson, M. Collins, B. Alonso, K. Cirone, F. Paolicchi, L. Gavey, M.T. Rahman, E. de Marchin, W. Van Praet, C. Bauman, G. Fecteau, S. McKenna, M. Salgado, J. Fernández-Silva, R. Dziedzinska, G. Echeverría, J. Seppänen, V. Thibault, V. Fridriksdottir, A. Derakhshandeh, M. Haghkhah, L. Ruocco, S. Kawaji, E. Momotani, C. Heuer, S. Norton, S. Cadmus, A. Agdestein, A. Kampen, J. Szteyn, J. Frössling, E. Schwan, G. Caldow, S. Strain, M. Carter, S. Wells, M. Munyeme, R. Wolf, R. Gurung, C. Verdugo, C. Fourichon, T. Yamamoto, S. Thapaliya, E. Di Labio, M. Ekgatat, A. Gil, A.N. Alesandre, J. Piaggio, A. Suanes, and J.H. de Waard. 2019. Control of paratuberculosis: who, why and how. A review of 48 countries. BMC Vet. Res. 15:198. doi:10.1186/s12917-019-1943-4.
